# On the Management of the Uterus after Parturition

**Published:** 1880-08

**Authors:** James H. Etheridge

**Affiliations:** Professor of Therapeutics and Medical Jurisprudence, Rush Medical College Chicago, Illinois


### Selections.

### ON THE MANAGEMENT OF THE UTERUS AFTER PARTURITION.

By JAMES H. ETHERIDGE, A.M,, M.D.,
Professor of Therapeutics and Medical Jurisprudence, Rush Medical College'
Chicago, Illinois.

I desire to call attention to a subject that every practitioner is interested in, and one which the general profession should use its powerful influence in revolutionizing the treatment of. That subject is, The Management of the Uterus After Labor. After devoting much time to considering this topic, especially in dispensary practice where one can see continually the lack of skillful care of the womb after lying-in, I have reached the solemn conviction that every woman, after delivery, should be under a physicians care for at least two months. Nearly all lying-in patients are discharged at the end of twenty or thirty days. Very many are not seen by the obstetrician after delivery. The general custom of remaining in bed for a space of seven or ten days after delivery is observed as a rule, and after that time the patient is allowed to go about and do what she ought not to do. The largest proportion of child-bearing women are poor and must labor. Household duties are resumed too early after delivery. Going up and down stairs, taking long walks, running sewing machines, even doing the usual week's washing and ironing and very many other kinds of labor are performed by the recently delivered women. The great strain thus put upon the female system after nine months of a most ceaselessly active and consuming tax upon her physical resources to maintain her own physiological balance, and at the same time to build up a distinct and separate human being within her, is a test of human physiological powers so unreasonable and prodigious that it constitutes a veritable outrage. Very many women endure this test and apparently are never worse by it, thanks to a wonderful recuperative power which their systems possess. A woman of feeble physique sees other women recover quickly from parturition, and attempts to do as her stronger sister has done, and the result is the inaugurating a strain of pathological processes within her pelvis from whose effects she promises never to be wholly freed. When too late, her mistake is discovesed, and she soon becomes a chronic invalid. Such a patient ought to be under the obstetrician's care at least two months after delivery.

Patients standing in need of this two months of the obstetrician's care are much more numerous than is generally supposed. Low down in the social scale we find the least number of women permanently crippled by their pregnancies and labors. In the middle walks of life very many more, relatively, are found, and in the upper classes of society it is almost the rule to find nearly all women who have been pregnant the victims, to some extent, of some disease of the pelvic viscera. It is an exceedingly common thing to find some patients among the last class who have been pregnant once or twice early in married life, and have lived barren for many years thereafter. It is as rare to find women in this class who have borne several children, as it is common to find multiparæ among the poorest classes.

The number of women constituting the so-called gynæcological cases treated to-day by physicians, who trace the commencement of their disorders back to some pregnancy, eventuating either in miscarriage or labor, is something surprising. To ascertain with some degree of satisfaction what proportion of these cases arise from miscarriages or deliveries, I have collated and tabulated the last 100 consecutive gynæcological cases, whose histories I have carefully preserved, in dispensary work. The question is always asked of those patients, "What is the cause of your trouble?" All of these cases are numerated below, and are classed under four heads. These cases are selected only to the extent of excluding virgins and women suffering from malignant and venereal diseases. I have found that the causes and diagnoses of these 100 cases can best be shown by arranging them in two columns, thus:

| | CAUSES, | | DIAGNOSES. | |
|---|---|---|---|---|
| 100 Gynæcological Cases. | Confinements | 50 | Hypertrophy | 34 |
| | Miscarriages | 28 | Uterine Catarrh | 53 |
| | Hard Work | 5 | Lacerated Cervix | 9 |
| | Unknown | 17 | Prolapsus | 2 |
| | | | Metrorrhagia | 1 |
| | | 100 | Retroflexion | 1 |
| | | | | 100 |

From this number it will be observed that 78 cases out of the 100 attribute their troubles to a certain confinement or miscarriage. In other words, 78 per cent. of gynæcological cases may be said to have originated immediately subsequent to gestation, partial or complete.

This brings me to a brief consideration of the management of women after labor. Given, a recently delivered woman, what is the physician's duty toward her? I am convinced more and more every year, from the lesson given by the interpretation of the foregoing briefly condensed syllabus, that the convictions of obstetricians will result in a new teaching, that a physician's duty to mothers will not be completed after delivery till the uterus has resumed its normal size. The time is near at hand when this treatment, preventive of gynæcological developments, will take rank with sanitary science, the most actively investigated field of medical science to-day.

Immediately after delivery the uterus presents a state of physiological activity equalled only by the process instituted in the same organ immediately after conception. "In no other structure do we ever see nutrition and growth going on so rapidly as here, where out of a mere mass of nucleated fibres and cells, an enormous body of numerous and well-marked muscular fibres becomes devoloped within the course of the nine months of pregnancy." After the completion of placental expulsion, the muscular fibres, exhausted by their prodigious action during labor, and so rendered prone to degenerate, and deprived also in a great measure of the supply of blood brought to them so profusely during gestation, undergo a fatty metamorphosis and almost all melt down and disappear. In normal cases the whole organ dwindles down and diminishes to nearly its original dimensions in from six to eight weeks. The muscular fibres are not absorbed as muscle, but, after first undergoing fatty degeneration, are absorbed as fat. This degeneration progresses from the inner to the outer layers. And right here, in this physiological process of degeneration and absorption, are presented the beginnings of so many cases of uterine disorder. Interference with this process develops disease. What constitutes this interference and how can the obssetrician avert it? These two questions as to cause and cure are what I wish to call special attention to. Clearly defining the first will suggest answers to the second question.

1. As to the cause.

It may be laid down as a broad principle that whatever lowers the health standard of a woman recently delivered, will retard or pervert the normal degenerative and absorptive processes herein considered. It is, of course, unnecessary to state that what may be called the health standard of such a woman is not the same as the health standard of a woman who has not so recently passed through with the immense s'rain and drain on her vital powers of building up, out of her own tissue and vitality, a new human being. Each one may be called a healthy woman in her way, and yet both are very far apart in very many particulars.

(*a*) Faulty nutrition lies at the basis of nearly or quite all cases of arrested uterine metamorphosis and absorption. Whatever contributes to this faulty nutrition is of the first importance. Lessened oxidation of the blood from diminished respiratory capacity in the last months of gestation, enfeebled circulation from the alleged fatty degeneration of the heart, incident to all pregnancies, perverted or exaggerated nervous manifestation, attending ingravidation, all contribute to lessen the digestive and assimilative functions in all women. The feebler the woman, ab initio, the more pronounced these contributions to faulty nutrition after delivery become, varying all the way from the minimum effect to the occasionally observed irretrievable impairment of health. The more that the obstetrician reflects upon these factors of faulty nutrition in detail and as a combination, the more he is astonished that more has not been written upon them, and the more is he surprised at the varying resistive capacities that women present at this time.

(*b*.) Another and but very little mentioned cause of faulty nutrition is diathesis. Patients presenting strongly marked rheumatic, strumous or gouty diatheses are extremely liable to become victims of a faulty nutrition, inducing arrest of fatty degeneration and absorption after delivery. Were time allowed, many cases illustrating the influence of diathesis in its deleterious effect upon women after labor could be cited. From the hundreds of recorded dispensary cases at my disposal, I could present multitudes of cases illustrative of the rheumatic and gouty diatheses in retarding involution. The confidence with which I can speak on this point is founded upon the results obtained from using so-called anti-rheumatic and anti-lithic remedies in treating and curing such patients.

(*c.*) Another factor of faulty nutrition is anæmia. This condition is so frequently produced by too abundant losses of blood, seen in obstetric practice, that merely mentioning it suffices to call to the mind of every gynæcologist numerous cases of involution arrested thereby. I know of no condition more common in gynæcological practice than the enlarged uterus in women suffering from anæmia dating from confinement.

(*d.*) Another condition contributing to faulty nutrition which ought to be dignified by special mention, although it is in fact a mere symptom, included in some of the aforementioned systemic conditions, and extreme frequent occurrence, is obstinate constipation. The multiform evils arising from this trouble are as familiar to physicians as household words. Yet there is no one thing more commonly overlooked and neglected by practitioners.

(*e.*) Another cause of faulty nutrition is laceration of the cervix uteri. This accident, resulting in cicatricial tissue, uterine displacement and obstructed circulation, speedily induces reflex, deleterious effects on the general nervous organism, which eventuate in a faulty nutrition of the system, and to this can we attribute the continuance of the enlargement of the uterus.

(*f.*) Another condition producing faulty nutrition is faulty hygiene, which includes, chiefly, bad air, improper food and insufficient protection against atmospheric changes.

(*g.*) Another condition conducing to faulty nutrition is the great activity of the depressing emotions. Great grief, fear, excessive anxiety, an excessively humiliated pride and the like produce such shocking devastation upon the system as to result in mal-nutrition.

(*h.*) Another cause of arrested involution is occasionally met with, which obstetricians should emphatically denounce, and that is the too early resumption of the sexual act.

(*i.*) Another cause of faulty nutrition, resulting in permanent uterine enlargement is that peculiar systemic condition characterized by a general depravity of all the powers of life, called neurasthemia. The more closely this term, as applied to the condition that it defines, is investigated, the more does it seem to be a *pons asinorum.* Occasionally the obstetrician, bewildered for an explanation, is justified in taking refuge behind this term to explain the cause of sub-involution.

What is the obstetrician's duty to the recently delivered woman? Evidently, to treat her till the uterus has resumed its normal size. To do this involves anything but routine treatment. Each case needs treatment indicated by itself. Any physician attempting to treat all cases alike will soon abandon such treatment and make each case an individual study. For the sake of convenience, the treatmant can be divided into two heads; first, general, and second, local.

First. The general treatment reduces the specialist to the rank of the generel practitioner, and involves taking into consideration every factor of health and disease. Above all, the patient must be put into possession of a digestive power as perfect as can be secured. The stomach and intestines must be made to do their individual work as vigorously as the conditions found will admit. Stomachic dyspepsia, tympanitis, costiveness, and constipation must be reduced to a minimum. The obstetrician's armamentarium against these symptoms is very great. Particularizing individual remedies would carry this paper to the proportions of a volume. It must suffice to say that every alimentary symptom must be carefully scrutinized and corrected.

General treatment also involves attention to the cutaneous and urinary functions.

Of infinite value to the obstetrician is a knowledge, well digested and made practical, of diathetic taints. These modifying conditions are so overshadowing in their influence upon the functions that nothing short of a speedy and intelligent recognition of them will make the practitioner's prescriptions, in most instances, aught but lucky ventures in correcting them.

Anæmia should be treated with ferruginous and bitter tonics. Produced, accompanied or modified by rheumatic, gouty or strumous diathesis, it demands remedies specially calculated to lessen such diathetic taint, before so called anti-anæmic remedies can be of signal advantage. Losing sight of this fact often results in defeat, easily avertable.

Neurasthenia must be treated in a way sui generis. Massage, aggressive tonics, the best of physical and mental hygienic conditions, food the most nutritious and in the largest quantities possible for the patient to digest and assimilate, and occasionally, electricity, seem to constitute the most approved treatment of to-day.

Second. The special treatment of women after confinement, where needed, consists chiefly in the use of means directed to the uterus itself and its accompanying organs. Each case furnishes its own indications.

Where lacerations of cervix or perineum exist they must be closed.

When the uterus is found too low down from its preternatural weight, it should be sustained by some pefectly fitting mechanical support. Any degree of prolapsus produces disordered uterine circulation, eventuating in engoregement, which, in turn, by encroaching upon the lymphatics, prevents the performance of their special functions.

When no cervical laceration exists and the uterine circulation is not interfered with by prolapsus from any cause whatever, a few measures of great efficacy may be used to hasten involution. The first one of these is ergot. The fluid extract ought to be frequently administered, in comparatively small doses—e. g. 10 to 15 drops, four or five times per day. When any contra-indication exists against its use by the stomach, nausea, vomiting, cephalalgia, or any diminution of the lacteal secretions, or untoward effect upon the babe or wakefulness or intestinal colic, it can be given by the rectum twice a day, in milk, in doses proportionally increased, or in suppositories, containing ergotine.

The second measure available is hot water injections. These, given per vaginam, as hot as can be borne, twice or thrice daily, in amounts varying from one to two gallons, are of extremely great efficacy in promoting involution. Very often they cannot be given so often in so large amounts. The patient is unabled to administer them without imposing an unwarrantable amount of fatigue herself. When she cannot receive the benefit of them used to their greatest advantage per vagina, very often a maximum of good can be derived by putting a quart of hot water into the rectum, a method recently advocated by Dr. Chadwick. Twice daily a rectum filled with hot water will produce signal benefit. The fact must not be lost sight of, however, that simple hot water may produce disturbance in the rectum by denuding the mucous membrane of its protective mucus and thereby setting up the commencement of a prochitis.

The explanation of the modus medendi of hot water is its well and long known powerful stimulant effect upon the vaso-motor constrictor system of nerves, producing a diminution of the caliber of blood vessels. In this way decongestion is quickly obtained as is comparatively easily secured.

Another measure of inciting the resumption of the act of involution in cases called chronic, is the use of tents for repeated distensions of the uterus. Slippery elm, tupelo or sea tangle can be used. The resort to these seems perfectly justifiable where the arrest of uterine metamorphosis soon after delivery seems to have assumed an obstinate phase.

Another measure that has been used by the advocates of manipulative treatment is massage—uterine massage. The American physician evidently needs educating in this measure, because it is an extremely easy matter to deride it and politely cough it down. The most incredulous physician needs but a few instances illustrative of the extremely rapid and satisfactory cure of this class of cases to satisfy him of the excellence of uterine massage and to make him an earnest advocate of it.

To Recapitulate: The aim of this paper is to contribute to the totality of our knowledge of the causes of gynæcological disorders, and to assist in creating opinion which shall eventuate in obstetric authorities imparting more correct and elaborate teaching as to the obstetrician's duties to women after confinement. It is less than thirty years since any definite opinion has been expressed by the authorities mentioned, as to how long a time the uterus requires to return to its natural proportions after its evacuation. Smelle claimed but three weeks and Scanzoni sixteen weeks for the period of normal involution. This difference of opinion arose from the lack of attention involving extended observation upon this subject. Let any physician, profoundly convinced that text books do not teach with sufficient emphasis the duty of the obstetrician to recently delivered women, as indicated by the result of dispensary observations in this particular, attempt to keep his post-puerperal patients under observation beyond the customary "theee days after delivery," and he will soon be brought face to face with the fact that the obstetric custom of to-day compels him, perforce, to be a prolific contributor to the myriads of gynæcology patients so surprisingly discovered in this generation.

Future works on obstetrics will be incomplete if they do not include definite and minute instructions as to the care of the postgravid uterus. They should teach that so long as the fundus uteri rises above the symphisis pubis, the patient should rest more than she exercises—that, when the progressive character of involution is arrested, the minutest attention to the patient's general condition is demanded, together with special measures to secure the continuance of a diminishing of the uterus to its normal size.

I. Among the causes of defective uterine involution may be included:

(*a.*) Faulty nutrition.

(*b.*) Diathetic taints.

(*c.*) Anæmia.

(*d*) Obstinate constipation.

(*e.*) Lacerations.

(*f.*) Faulty hygiene.

(*g.*) Depressed emotions.

(*h.*) The marital act.

(*i.*) Neurasthenia.

II. The treatment recommended embraces:

1. *General Treatment:*

(*a.*) Correction of all alimentary derangements.
(*b.*) Removal of anæmia.

(*c*) Neutralizing the effects of diathetic taints.

2. *Special Treatment:*

(*d.*) Closing of lacerations and using of needed mechanical supports.

(*e*) Ergot by stomach or rectum.

(*f.*) Hot water injections per vaginam vel rectum.

(*g.*) Dilatation by using tents.

(*h.*) Uterine massage.

Dr. Jackson—I regard the paper as one of very great interest and full of important suggestions. The statement, however, that more than 75 per cent. of our gynæcological cases are dependent uyon sub-involution of the uterus or any other result of parturition which should be a physiological process, is, to say the the least, a startling one; and, if it be correct, the fact shows the great importance of adopting proper measures for the prevention of such consequences. In a general way I can endorse the treatment advised by the essayist for this purpose; attention to the state of the bowels, the kidneys, skin, the timely use of ergot, etc.

I have found ergot especially useful in cases where the uterus is enlarged and still soft in texture before the indurated stage is reached. Arsenic is also useful in these cases. In those of long standing, where the uterus is hard and enlarged, I have frequently applied the fuming nitric acid with excellent result.

The hot water douche, while freqently an excellent and useful remedy, is not always so. The blanching of the vagina and vaginal portion of the uterus produced by its employment is not of long duration. I have seen these parts a half hour after being thus blanched become fairly purple from the congestive return of blood. Then, too, some persons suffer so much from flushings of the face and headache after its use as to make it unsuitable remedy at least for them

Dr. Earle said that if the figures presented to us were correct, the accidents and bad results incident to child-bearing must be far greater among the poor than among all other classes. In a practice of ten years, mainly among the middle class, with a moderate percentage of the upper or more wealthy part of the people, not two per cent. had ever applied for any uterine treatment of which child-bearing was thought to be the cause. The surroundings of the lower class, and the kind of food which, from necessity, they must take, had much to do with producing the condition described by the essayist. At least four of the symptoms enumerated were due to hard work and poor food, namely, bad nutrition, diathesis, anæmia and constipation, and it hardly seems necessary for me to refer them to child-bearing.

Two important causes of disease peculiar to women were prevalent among the very poor, and in many cases it was impossible for the physician to do away with these causes. I refer particularly to too early getting up after confinement, and secondly to the debility following a long course of lactation where there is insufficient food with really more work than any nursing mother should be allowed to perform.

I am an advocate for at least two full weeks' rest in bed for every lying-in woman. I know, however, that it is impossible for many to give this time; and then there are those who oppose it—certain nurses and a few physicians who seem to think it is the best procedure—indeed, the creditable thing—to get a woman out of bed the second or third day after delivery. I believe it to be alsolutely wrong to encourage this, for I am sure it gives rise to many of the symptoms to which our essayist has called our attention. And further, lactation among some of these women, especially those who are obliged to work very hard, and from pecuniary causes are unable to have a varied and generous diet, is frequently followed by these same symptoms.

Later in the discussion, Prof. Miller took exception to what he understood to be Dr. Earle's opposition to mothers nursing their children.

Dr. Earle explained that he did not oppose lactation—indeed, always favored it. Art could not supply a substitute; yet with insufficient food for the mother, in addition to hard work, it did seem to him that debility, prostration and anæmia frequently occurred without the factor of child-bearing entering as a cause.

Dr. Etheridge: The objection to hot water injections producing temporary anæmia, followed by increased turgescence, holds good only where occasional injections are used, small in amount. When they are resorted to three or four times every twenty-four hours, to the extent of six or eight quarts each, the blanching effect of one injection remains till the next one is used. Hence frequent large injections are recommended.

Attention to the alimentary functions is of the highest importance. The tendency to portal engorgement, with its resultant anorexia, stomachic and intestinal indigestion and constipation, can be best removed in most cases by the use of laxative—not cathartic—doses of mineral waters. An extensive use ,of the various waters has resulted in a preference for the "Ofner Rakoczy Bitterwater." A wineglassful in one-third or one-half a cup of warm water or warm milk in the empty stomach mornings, will produce an astonishing effect in quickening a sluggish portal circulation, removing effete products (and thereby becoming a veritable "blood purifier") and in invigorating the appetite and the secretions of the alimentary secernents. The result of thus using the Rakoczy water is a digestion of more food, and the elaborating of more healthy blood, which is the only ground-work of permanently correcting faulty nutrition. The dose must be increased or diminished to suit each indiviual case, the end sought being a natural, daily fecal evacuation, without nausea or griping. Calling this water by its other name—a "blood purifier" —removes a popular objection to daily catharsis. Continuing its use for six weeks will result in a new order of portal nutrition which does not demand its continuance.

It is a curious clinical fact that where an active purgation is demanded, the color of the feces indicates a definite distinction of choice between mercury and podophyllin; where the dejections are too light, mercury is followed by the best results. When they are too dark, podophyllin should be used. Physiology teaches that between three and four pints of bile are poured into the duodenum every twenty-four hours. Nearly all of this bile is re-absorbed, passes through the liver, is re-excreted and again passed into the bowel. Day after day and week after week throughout life the liver is incessantly active, secreting and excreting. Were this hepatic function checked to any great extent, dire disaster would result. It is a gross error to hold that "the liver is inactive"—"is dormant," etc. The fact is, that, like the kidneys and heart, it never sleeps till death supervenes. What results when a "cholagcgue cathartic" is given, followed by "bilious discharges," is, the stepping in between the progress of the bile along the intestine and its re-absorption, and preventing the latter. As a result, this fluid is hurried along the canal, and it makes its appearance in the dejecta. The mercury or podophyllin does its good work by removing thus old bile practically worn out, and hence an excretion which ought to be removed, and enables the liver to purify the general circulatory torrent by eliminating from it the biliary constituents, which in excess cause the so frequently observed condition known as icterus. In this way the obstetrician can contribute to removing "faulty nutrition" in post-puerperal patients.

A lacerated perineum ought to be closed immediately after delivery. A lacerated cervix ought to be closed as soon as it is evident that involution is arrested. Where involution is complete, the propriety of operating for cervical laceration becomes a debatable question.

The use of ergot may be resorted to from delivery, and continued or suspended *pari passu* with the slowness or promptness of involution.

The objection that the 78 per cent. alluded to is applicable more to dispensary than to private practice, and that this proportion is too high because it excludes virgins, is difficult to substantiate, because in a city—*e. g.*, Chicago—it is easy to ascertain how many deliveries at term occur, and it is absolutely impossible ever to know how many abortions occur in "virgins" as well as in married women. It is as impossible to know how many gynæcological patients there are who never consult physicians for their diseases, and yet are veritable sufferers from uterine disease arising from defective involution.

Another item for contemplating in the syllabus given is the 17 per cent. of causes headed "unknown." Could the facts be known about each one of those seventeen cases, it would probably be found that the majority arose from defective involution. And it would in all probability be more nearly correct to maintain that the 78 per cent. should be brought up to the neighborhood of 90 per cent.

The subject of rest alluded to by Professor Miller is of such acknowledged importance that but one opinion concerning it can be entertained. I fail to appreciate the ambition manifested by some practitioners to have lying-in women get up early after confinements. It is impossible for any one to appreciate the intra-pelvic changes after delivery without deciding in all cases that the only safe course is to advise more of rest than exercise so long as the fundus rises above the pubic arch. Ten days ought to be a minimum time for a patient to remain in bed after delivery. I always advise a woman to remain in bed three weeks rather than ten days.

